# Broad-Scale Genetic Diversity of *Cannabis* for Forensic Applications

**DOI:** 10.1371/journal.pone.0170522

**Published:** 2017-01-20

**Authors:** Christophe Dufresnes, Catherine Jan, Friederike Bienert, Jérôme Goudet, Luca Fumagalli

**Affiliations:** 1 Laboratory for Conservation Biology, Department of Ecology and Evolution, Biophore Building, University of Lausanne, Lausanne, Switzerland; 2 Centre Universitaire Romand de Médecine Légale, Chemin de la Vuillette 4, Lausanne, Switzerland; 3 Department of Ecology and Evolution, Biophore Building, University of Lausanne, Lausanne, Switzerland; Universita degli Studi di Siena, ITALY

## Abstract

*Cannabis* (hemp and marijuana) is an iconic yet controversial crop. On the one hand, it represents a growing market for pharmaceutical and agricultural sectors. On the other hand, plants synthesizing the psychoactive THC produce the most widespread illicit drug in the world. Yet, the difficulty to reliably distinguish between *Cannabis* varieties based on morphological or biochemical criteria impedes the development of promising industrial programs and hinders the fight against narcotrafficking. Genetics offers an appropriate alternative to characterize drug *vs*. non-drug *Cannabis*. However, forensic applications require rapid and affordable genotyping of informative and reliable molecular markers for which a broad-scale reference database, representing both intra- and inter-variety variation, is available. Here we provide such a resource for *Cannabis*, by genotyping 13 microsatellite loci (STRs) in 1 324 samples selected specifically for fibre (24 hemp varieties) and drug (15 marijuana varieties) production. We showed that these loci are sufficient to capture most of the genome-wide diversity patterns recently revealed by NGS data. We recovered strong genetic structure between marijuana and hemp and demonstrated that anonymous samples can be confidently assigned to either plant types. Fibres appear genetically homogeneous whereas drugs show low (often clonal) diversity within varieties, but very high genetic differentiation between them, likely resulting from breeding practices. Based on an additional test dataset including samples from 41 local police seizures, we showed that the genetic signature of marijuana cultivars could be used to trace crime scene evidence. To date, our study provides the most comprehensive genetic resource for *Cannabis* forensics worldwide.

## Introduction

*Cannabis* is one of humanity’s oldest cultivated plant. It is thought to have originated in central Asia and was domesticated as early as 8 000 BP for food, fibre, oil, medicines and as an inebriant. This crop was since distributed across the world during the last two millennia and, due to its recent legalization in several countries, is increasingly exploited by several industrial sectors (hemp) and as a recreational drug (marijuana). The taxonomic status of *Cannabis* has always been disputed, as it encompasses multiple cultural, geographic, historical and functional aspects (reviewed in [1–4]). Whereas most authors now consider it a monotypic panmictic taxon, *Cannabis sativa*, three species or subspecies (*sativa*, *indica* and *ruderalis*) are often mentioned but without a comprehensive taxonomic grouping so far. The nomenclature may thus differ depending on whether it refers to morphological or chemical variation, geographic distribution, ecotype, as well as crop-use characteristics and intoxicant properties resulting from human selection [4–7]. *Cannabis* presumably diversified following selection for traits enhancing fibre and seed production (”hemp”) or psychoactive properties ("drug"). Importantly, *Cannabis* types differ in their absolute and relative amounts of terpenophenolic cannabinoids, notably Δ^1^-tetrahydrocannabinol (THC), the well-known psychoactive compound of marijuana, and the non-psychoactive cannabidiol (CBD). In this context, drug-type *Cannabis* (marijuana) is broadly characterized by a higher overall cannabinoid content than fibre-types. However, the most widely recognized criteria to assign a *Cannabis* plant to either “drug” or “hemp” type is the THC:CBD ratio, according to which three main chemical phenotype (chemotype) classes are recognized: hemp-type plants with a low ratio (THC:CBD < 1), drug-type plants with a high ratio (THC:CBD > 1), and intermediate-type plants with a ratio close to one [6, 8]. The informal designation *sativa* and *indica* may have various, controversial meanings. Morphologically, the name *sativa* designates tall plants with narrow leaves, while *indica* refers to short plants with wide leaves. Among the marijuana community however, *sativa* rather refers to equatorial varieties producing stimulating psychoactive effects (THC:CBD ≈ 1), whereas *indica*-type plants from Central Asia are used for relaxing and sedative drugs (THC:CBD > 1) [8].

The commercial interest for *Cannabis* has declined during the XX^th^ century due e.g. to the development of synthetic fibres and the stringent policies regarding its exploitation, but this iconic weed is recently regaining attention in many countries for its high medicinal, industrial and agricultural potentials (reviewed in [9]). However, its usage is still controversial, in particular from agro-economic, public health and forensic perspectives. Due to its intoxicant properties, the cultivation and possession of *Cannabis* is under strict legal regulations. High-THC:CBD varieties are prohibited in many countries but remain the most frequently-used illicit drug worldwide [10] (~180 million consumers in 2013, [11]), in the form of marijuana (dried inflorescences) or hashish (resin). In contrast, low-THC:CBD hemp crops can be exploited under licensed control for seed oil, fibres and pharmaceutical industries. For instance, quantitative measures of THC-content are currently considered by the EU for approval as a licensed hemp cultivar (below 0.2% THC weight per weight in the mature dry inflorescences; http://ec.europa.eu/food/plant_en). Yet, hemp and marijuana varieties are hardly distinguishable morphologically and discrimination of drug *vs*. non-drug chemotypes by quantitative THC-dosage has also proven inadequate due to its dependence on environmental factors, to the strong variation during the plant’s life cycle, as well as between individual plants [12–13]. In addition, the qualitative assessment of THC:CBD ratio is also problematic for an unequivocal discrimination between fibre and drug types, due to the presence of a largely variable intermediate chemotype class, the occurrence of several exceptions (e.g. hemp accessions with a THC-predominant chemotype; [14–16]) and the common practice among drug breeders to produce hybrid varieties.

This issue largely impedes crops’ improvement and full-scale industrial development; it even causes a security risk, as licensed crops may be used as a cover for illegal drug production. Moreover, it significantly limits the ability of law-enforcement agencies to trace drug seizures and link illegal producers to organized crime syndicates supplying the black market of *Cannabis* drugs. In addition, *Cannabis* can have long-distance dispersal capabilities [17], and fibre crops might face cryptic contamination by pollen from drug varieties.

Genetic tools offer a promising avenue to overcome these issues, especially to distinguish between drug *vs*. non-drug plants [18]. Importantly, genetics requires small amounts of tissues as a DNA source, whereas chemical analyses necessitate inflorescences. A promising aspect has been to genotype loci directly linked to THC synthesis [8, 19] in association with chemotype profiling. However, this association is not ubiquitous [14–15], and genotyping may be compromised by complex gene duplications, pseudogenes [20–22] and, that only a limited number of varieties among the tremendous *Cannabis* diversity has been validated [15]; moreover, chemotype seem to greatly vary even among genotypes [20].

A parallel, complementary approach is to discriminate drug *vs*. hemp plants from their non-adaptive genetic variation. Until the recent past, the genetic diversity of *Cannabis* has remained surprisingly under-investigated, partly due to the important restrictions imposed by anti-drug policies, even for scientific inquiries. In the last few years, a draft genome of *Cannabis* was published [22] and high-density Single-Nucleotide-Polymorphism (SNP) data obtained from Next-Generation-Sequencing (NGS) techniques evidenced genome-wide differentiation between hemp and marijuana plants [23]. However, genetic resources applicable for forensics remain under-developed. Forensic investigations require sets of sufficiently informative loci that can be genotyped in large batches of samples in a rapid and affordable manner, such as microsatellites (Short-Tandem-Repeats, STRs). Another prerequisite is that the species’ diversity is exhaustively represented in reference databases, both within and among varieties, so that investigated samples of unknown origin can be identified with statistical confidence. In *Cannabis*, these two aspects are challenging given the diversity of varieties, their complex breeding histories, as well as the rapid shifts of the drug varieties available on black markets. In addition, hemp and marijuana diverged during the human era and still largely share a common pool of genetic variation [23].

Several microsatellite analyses were previously performed on *Cannabis*. Some loci became available in the early 2000s [24–26] but remained scarcely tested at the individual or population level. The first STR multiplex kit for forensics was validated years later [27], and subsequently trialed to distinguish fibres from confiscated drug seizures in Australia, with moderate success [28]. Another STR kit was developed by Köhnemann et al. [29], although without reference data. Using transcriptomic sequences (EST), Gao et al. [30] isolated >100 STRs, allowing them to discriminate between Chinese and European hemp samples according to their geographic origin. Other studies genotyped *Cannabis*, notably from police seizures, using new or published markers [31–35]. However, although these studies are regionally and timely relevant, they rely on limited sample sets (i.e. few varieties and few individuals per variety, and/or only representing plants available on a regional black market at the time of confiscations), thus hardly accounting for the different levels of genetic variation of *Cannabis* stocks. So far no comprehensive database of *Cannabis* diversity exists for broad-scale forensic enquiries.

Considering these limitations, we developed a new STR resource for *Cannabis* forensics. We analyzed intra- and inter-populational variation at 13 published STRs markers in >1 300 *Cannabis* samples from 48 fibre and drug accessions, broadly representative of known hemp and marijuana varieties (S1 Table), and characterized unknown samples of various origins, notably police seizures. We aimed at (i) showing that these loci fully recover the genetic structure between marijuana and hemp; (ii) demonstrating that anonymous samples can be confidently assigned to either plant types; and (iii) documenting the genetic diversity among and within samples and its potential for forensic investigations.

## Results and Discussion

**Fig 1:**
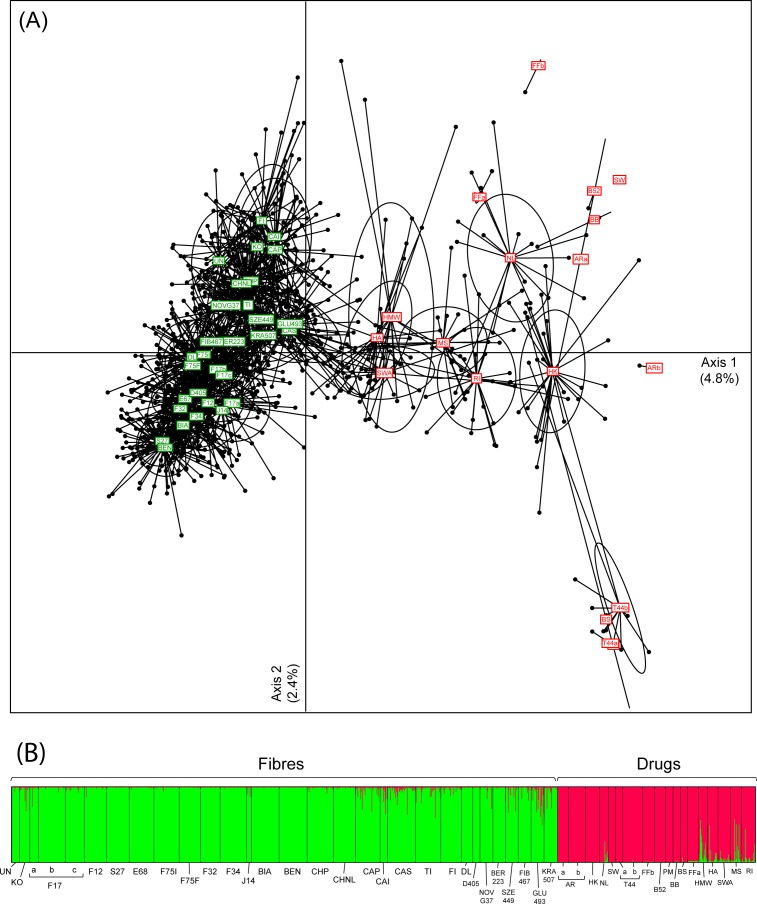
**Principal Component Analysis (A) and Bayesian clustering with STRUCTURE (B) of individual genotypes from 48 *Cannabis* accessions.** Fibre and drug accessions are displayed in green and red respectively on the PCA. Ellipses illustrate 80% inertia of each accessions. Dots represent individuals, linked to their accessions (labelled within colored squares). On the STRUCTURE barplots, colors show the probability of assignment to each cluster (K = 2), perfectly distinguishing fibres from drugs.

The selected STR markers (detailed in S2 Table) unanimously recovered the strong structure between fibres and drug *Cannabis* samples. This is clearly depicted by a Principal Component Analysis (PCA, Fig 1A), genetic distances between accessions (F_st_, S1 Fig) and genotype clustering by STRUCTURE (Fig 1B), where two groups appears as the best clustering solution (ΔK_2_ = 1205.6). As recently evidenced from NGS data [23], this pattern reflects differentiation between hemp and marijuana over the entire genome, not only at genes underlying THC and fibre synthesis. Some drugs and fibres show weak signs of genetic admixture (intermediate PCA scores and STRUCTURE probabilities, Fig 1; lower F_st_, S1 Fig), which might stem from introgressive crossbreeding, as reported elsewhere [23]. Interestingly, except for RI (*indica*/*ruderalis* hybrid), all drug varieties closely-related to hemps are of *sativa* ancestry (HMW, HA, SWA, MS; based on available information from suppliers). This would support the common assumption that hemp varieties selected for fibre and seed production derived from *sativa*, although this view has been challenged by other studies that found more similarities between hemp and *indica* [7, 23, 36]. Alternatively, *sativa* drugs, which are nowadays distributed in more equatorial regions, may be frequently crossbred with *indica* and agricultural varieties to facilitate their cultivation in temperate countries. In any case, marijuana genetic diversity seems weakly associated with the documented breeding history: we also performed a PCA solely on drugs, which only marginally clustered according to their main *sativa* and *indica* pedigree (S2 Fig). Some cultivars of the same appellation appear genetically distinct (e.g. Alpine Rocket, ARa and ARb, F_ST_ = 0.36) whereas others harboring different names are genetically identical (e.g. PM, T44, BS, F_ST_ = 0.00; identical clones shared by ARa and B52, S1 Table). Overall, these observations are in line with the general conclusions of Sawler et al. [23] that drug varieties are often misinformed due to the clandestine nature of *Cannabis* breeding over the last century, and that names do not necessarily reflect a meaningful genetic identity. In addition, hemp varieties grouped according to reproductive characteristics, as expected (dioecious versus monoecious; S1 Table), as a result of their breeding history (illustrated on the PCA, Fig 1; F_st_ tree, S1 Fig).

**Fig 2:**
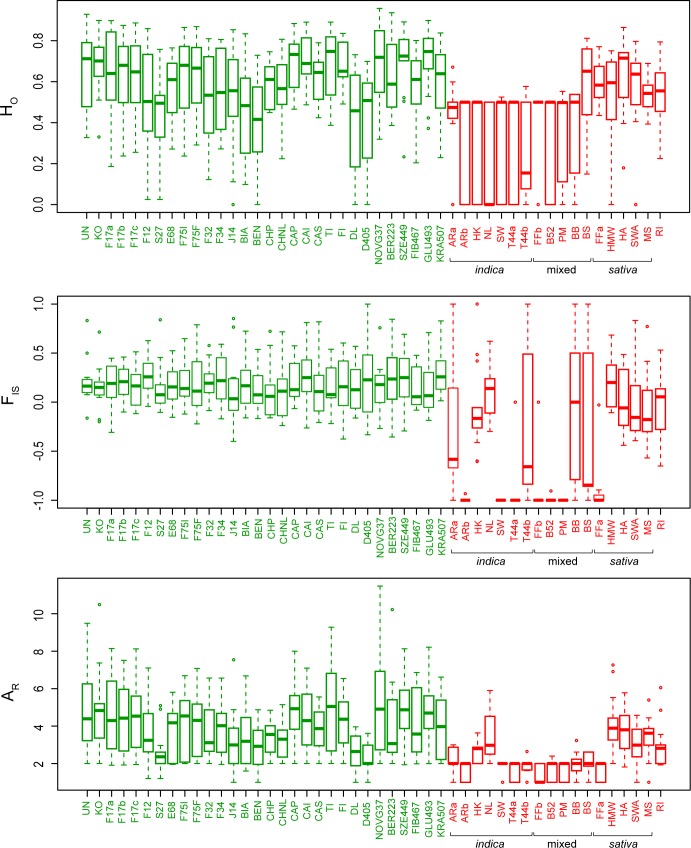
Genetic diversity within each *Cannabis* accession. F_IS_: inbreeding coefficient; H_O_: observed heterozygosity; A_R_: allelic richness (scaled for 8 individuals). For drugs, main documented *sativa*/*indica* component are indicated.

Intra-variety diversity was relatively similar among hemps (Fig 2). Allelic richness (average number of alleles per population A_R_, scaled to eight individuals) and heterozygosity (H_O_) averaged 4.0 ± 0.8 and 0.59 ± 0.10 respectively (Fig 2). All varieties had positive inbreeding coefficients (F_IS_ = 0.19 ± 0.05), potentially reflecting bottlenecks linked to current breeding practices. The overall differentiation among hemps was relatively low (F_ST_ = 0.15 ± 0.07; S1 Fig). In contrast, marijuana featured lower diversity within varieties (A_R_ = 2.3 ± 0.9, H_O_ = 0.41 ± 0.15; Fig 2) but substantially higher genetic distances among them (F_ST_ = 0.39 ± 0.16; S1 Fig). We detected identical genotypes (clones) and strong excess of heterozygosity among several breeds (all of *indica* or mixed origin, S1 Table), which translates into A_R_ of 2, H_O_ of 0.5 and F_IS_ reaching -1 (Fig 2), resulting from clonal breeding from hybrids of two different parental strains. Interestingly, *sativa* drugs featured more hemp-like patterns of diversity. Overall, the homogeneous gene pool of hemps suggests more frequent crossbreeding compared to drugs [23], especially of *indica* content, and/or that a wider genetic base has been sourced by the hemp industry. Marijuana is often propagated clonally for practical reasons as well as to protect the genetic identity of varieties from contamination by wind-dispersing pollens, thus reducing diversity and triggering strong heterozygosity in F1 cross-breeds. Moreover, all *Cannabis* drug forms are dioecious, and males, which produce lower amounts of THC than females, are discarded by breeders, which further reduces diversity.

The diversity captured by our STR markers appears well representative of the genomic background of *Cannabis*: our results are overall very concordant with high-density SNP data [23]. Our STR database thus seems appropriate for broad-scale forensic applications, in particular to discriminate between drug *vs*. non-drug samples, one of the main task of *Cannabis* forensics. To demonstrate this ability, we performed genetic assignment tests (direct or resampling-based) on random subsets of drug and fibre samples, using the remainder of the dataset as reference (detailed in the Methods section). The direct test always correctly assigned every sample to their plant type (Table 1). The more conservative resampling approach never misassigned any specimen (Table 1). Many individuals are yet not assigned to any group (even the correct one) because genotypes are considered not statistically informative enough by this conservative analysis. We further evaluated the database by genotyping 340 additional *Cannabis* samples of various origins (bird food, drug and fibre specimens, uncertain industrial cultivars and police seizures). Known specimens (n = 8) were all correctly assigned with high confidence (Table 2). All but one industrial cultivars (n = 37) consisted of hemps, with few getting assignment probabilities below 0.95 (Table 2). Confiscated samples (n = 295, from 41 different seizures) could be unambiguously assigned except for three specimens (Table 2).

**Table 1:** Database auto-evaluation by assignment tests of random subsets of fibre and drug samples. Values indicate the probabilities *P* of assignment (direct method) and inclusion to either groups (resampling method), as well as their standard deviations among replicate subsets (n = 10).

	Direct method	Resampling-based method	
	Probability of assignment to the correct group	Probability of inclusion	
		to fibres	to drugs
Fibres	1.00 ± 0.00	0.50 ± 0.03	0.0 ± 0.0
Drugs	1.00 ± 0.00	0.0 ± 0.0	0.53 ± 0.04

**Table 2:** Assignment trial (direct method) of 340 test samples from known (bird food, known fibres and drugs) and unknown nature (industrial cultivars and police seizure). We considered assignments “safe” where the probability of assignment *P* was above 0.95.

		Mean probability of assignment		Number of safe assignments (*P* > 0.95)
	n	to fibres	to drugs	
bird hemp seed	1	1.00	0.00	all
known fibres	5	1.00	0.00	all
known drugs	2	0.00	1.00	all
industrial cultivars (hemp)	36	0.99	0.01	33 (92%)
industrial cultivars (marijuana)	1	0.00	1.00	all
police seizures (hemp)	13	0.99	0.01	all
police seizures (marijuana)	282	0.00	1.00	279 (99%)

These results clearly illustrate the relevance of our new database for forensics. Notably, it outperforms the reference published by Howard et al. [28] for Australian seizures, which suffered substantial mis-assignment risks, but yet so far was the only available resource properly tested by statistical assignments. Moreover, compared to previous studies, our sampling scheme has the advantage of covering a broad range of *Cannabis* varieties and accounts for their intra-variety variation. The latter seems important to consider, as some marijuana (*sativa*) and hemp cultivars share closely-related gene pools, sometimes making their discrimination difficult.

In addition, the strong genetic structure between drug cultivars may provide opportunities for police investigations of narcotrafficking. One challenge for law-enforcement agencies is to trace evidences collected at crime scenes in order to connect and convict acting members of crime syndicates. Most marijuana individuals/germlines show unique genetic profiles at our markers (Fig 1A, S2 Fig), so they could be suitable for this task. We screened for identical genotypes among the seized Western-Swiss samples of our test dataset, where the probability of identity P_I-sib_ is 8.9 × 10^−5^. We could established five groups of related seizures (some even matched by several germlines) thus with 99.991% confidence (S3 Table); the remaining 25 seizures were genetically different (S3 Table). Given the high resolution at such narrow regional scale, this approach could also be applied at national or international levels. The illegal trade of *Cannabis* is one of the most developed illicit industries in the world (> 7 000 tons seized in 2013, [11]), yearly generating enormous profits used to finance other criminal activities. Exploiting the genetic heterogeneity of marijuana should be the focus of further forensic development to aid the international fight against narcotrafficking.

To date, our STR database is the most powerful resource suitable for routine forensic analyses of *Cannabis*. Yet, it remains limited by several aspects. First, drug *vs*. non-drug discrimination can be ambiguous for some samples, given the lack of differentiation and/or crossbreeding practices between few hemp and marijuana varieties. Second, the plant type of our reference samples rely on the information provided by the suppliers, which could be confirmed by chemotyping analyses. Third, more sensitive applications such as tracing drug evidences might require a finer resolution. In both cases, updating the database with additional markers and reference populations, especially new drug varieties, seems a worthy investment. Further development would benefit from international collaborations. An array of genetic studies have been conducted on *Cannabis* in just a few years by different research teams (see Introduction), each contributing specific sets of samples and markers. Given the tremendous diversity of marijuana and the legal difficulty to access samples, joint efforts between *Cannabis* genetics’ experts worldwide would allow unprecedented opportunities to extend forensic advances and promote the development of the industrial and therapeutic potential of this emblematic species.

## Materials and Methods

### Ethics statement

This study does not involve any endangered or protected species.

### Sample collection

We built a collection of 1,324 *Cannabi*s samples from 30 accessions of fibres (n = 972 from 24 different varieties) and 18 accessions of drug (n = 352 from 15 varieties). These accessions broadly cover the legal European hemp varieties (landraces, cultivars selected from landraces and cross-bred cultivars) and marijuana diversity (identified a priori as *sativa*, *indica* and hybrids by breeders). In order to also capture intra-variety variation, we included large population samples for each accession (27 samples on average, from 9 to 50). Seeds and leaves were obtained from agronomic companies, germplasm collections, police seizures or commercial stores; seeds were germinated at the University of Lausanne (Switzerland). S1 Table provides sample origin and reported breeding history, given available documentation and information provided by the suppliers.

To evaluate our reference database, we further considered 340 additional test samples from uncertain (police seizures, industrial cultivars) or known types (fibre and drug samples not included in the reference database). Confiscated plants (n = 295) represented 41 police seizures across Western Switzerland from 2005 to 2010. Details are provided in S3 Table.

### DNA extraction and microsatellite genotyping

DNA was extracted from approximately 25 mg of dried plant leaves using the FastDNA Kit (Qbiogene, Carlsbad, CA) following the manufacturer’s instructions. Thirteen published microsatellite loci were analyzed [24–25], including the ten from Howard et al.’s forensically validated kit [28]. DNA amplifications were performed according to their STR multiplex system (M1 and M4), slightly modified to include ANUCS202 to multiplex M4. In addition, we integrated a new multiplex M5 to amplify loci ANUCS201 and H09-CANN2. Detailed information on markers and multiplexes are available in S2 Table. PCR conditions were as follows: 95°C for 5 min (initial denaturation); 10 cycles consisting of 30”at 95°C, 30” at 66°C down to 54°C (-3°C/2 cycles) and 45” at 72°C (top-down PCR); 30 regular PCR cycles consisting of 30” at 95°C, 30” at 50°C and 45” at 72°C; 90”at 72°C (final elongation). Amplicons were run on an ABI PRISM 3130 Genetic Analyzer (Applied Biosystems) and genotyped were scored using GeneMapper v3.2 (ABI).

### Population genetic analyses

We analyzed the genetic structure and diversity of *Cannabis* by three different approaches. First, we performed a Principal Component Analysis (PCA) on individual genotypes using the R packages ade4 and adegenet [37]. Second we conducted Bayesian clustering of genotypes into groups with STRUCTURE [38]. We used the admixture model without prior on sample origin, and tested from 1 to 11 groups (K), with 10 replicates per K. Each run consisted of 100’000 iterative steps following a burn-in of 10’000. We applied the Evanno method [39] to determine the most likely number of groups summarizing the data, as implemented in STRUCTURE HARVESTER [40]. Replicates were combined using CLUMPP [41] and graphical displays of admixture proportions (barplots) were built with DISTRUCT [42]. Third, we conducted population-based analyses with FSTAT [43], by calculating pairwise genetic distances between accessions (F_ST_) as well as the following diversity indices for each accession: observed heterozygosity (H_O_), inbreeding coefficient (F_IS_) and allelic richness (A_R_, scaled to 8 individuals).

### Genotype specificity and assignment tests

We used GenAlEx 6 [44] to compute, within and among accessions, the number of private alleles (P_A_) and probabilities of identity (P_I_), i.e. the probability to have identical genotypes by chance. For the latter, we considered the conservative estimate of P_I-sib_ when the data potentially includes siblings, as appropriate for *Cannabis* samples. We also used GenAlEx to match identical genotypes, notably to identify clones (function “match”).

To assess the power of discrimination between hemp and drug types, assignment analyses were performed with GeneClass2 [45]. First, we auto-evaluated our database by assigning ten re-sampled random subsets (representing about 10% of the total dataset, n = 100 for fibres, n = 40 for drugs) using the rest of the data as reference. To this end, two different methods proposed by the software were applied, using Bayesian criteria [46]. The first approach (direct method) estimates the proportion of correctly assigned samples to the most likely population of origin. The second approach (resampling-based method) computes the probability that samples belong to each reference population, and aims at minimizing the risk of mis-assignment, i.e. when individuals feature genotypes that can occur in the “wrong” reference population (type I error). This was achieved by simulating the likelihood distribution of 10,000 independent genotypes, for each reference population (with a Monte-Carlo resampling algorithm, [47]), against which the genotypes to assign can then be compared. Rejection or inclusion is then decided upon a threshold (*P* < 0.01). This approach does not assume that all source populations have been sampled. Second, we assigned (direct method), our 340 test samples, which consist mostly of unknown varieties.

## Supporting Information

S1 Fig Tree of genetic distances (pairwise F_st_) between *Cannabis* accessions. Monoecious hemp are highlighted in grey.
(TIF)

S2 Fig Genetic structure among marijuana samples. (TIF)

S1 Table List and details on the *Cannabis* accessions. (XLSX)

S2 Table List and details on the STRs markers. (XLSX)

S3 Table List and details on test samples. (XLSX)

## References

[1] Small E, Cronquist A. A practical and natural taxonomy for *Cannabis*. Taxon. 1976; 25: 405–435.

[2] Clarke R, Merlin M. *Cannabis—*Evolution and Ethnobotany: University of California Press; 2013.

[3] Small E. Evolution and classification of *Cannabis sativa* (Marijuana, Hemp) in relation to human utilization. Bot Rev. 2015; 81: 189–294.

[4] Welling MT, Shapter T, Rose TJ, Liu L, Stanger R, King GJ. A belated green revolution for Cannabis: virtual genetic resources for fast-track cultivar development. Front Plant Sci. 2016; 7:1113 10.3389/fpls.2016.01113 27524992 PMC4965456

[5] de Meijer EPM, van Soest LJM. The CPRO Cannabis germplasm collection. Euphytica 1992; 62: 201–211.

[6] de Meijer EPM. The Chemical Phenotypes (Chemotypes) of Cannabis In: Pertwee RG, editor. Handbook of *Cannabis*. Handbooks in Psychopharmacology: Oxford University Press; 2014 Pp. 89–110.

[7] Hillig K. Genetic evidence for speciation in *Cannabis* (Cannabaceae). Genet Resour. Crop Evol. 2005; 52: 161–180.

[8] Hillig KW, Mahlberg PG. A chemotaxonomic analysis of cannaboid variation in *Cannabis* (Cannabaceae). Am J Bot. 2004; 91:966–975. 10.3732/ajb.91.6.966 21653452

[9] Andre CM, Hausman JF, Guerriero G. *Cannabis sativa*: the plant of the thousand and one molecules. Front Plant Sci. 2016; 7:19 10.3389/fpls.2016.00019 26870049 PMC4740396

[10] Anderson P. Global use of alcohol, drugs and tobacco. Drug Alcohol Rev 2006; 25: 489–502. 10.1080/09595230600944446 17132569

[11] United Nations Office on Drugs and Crime. World Drug Report 2015 (United Nations publication, Sales No. E.15.XI.6). Available: https://www.unodc.org/documents/wdr2015/World_Drug_Report_2015.pdf

[12] Rowan MG, Fairbairn JW. Cannabinoid patterns in seedlings of Cannabis-sativa L and their use in determination of chemical race. J Pharm Pharmacol. 1977; 29: 491–494. 19599 10.1111/j.2042-7158.1977.tb11375.x

[13] Baker PB, Gough TA, Taylor BJ. The physical and chemical-features of Cannabis plants grown in the United-Kingdom of Great-Britain and Northern-Ireland from seeds of known origin. Bull Narc 1982; 34: 27–36. 6291677

[14] Welling MT, Liu L, Shapter T, Raymond CA, King GJ. Characterization of cannabinoid composition in a diverse *Cannabis sativa* L. germplasm collection. Euphytica 2016; 208:463–475.

[15] Stagginus C, Zörntlein S, de Meijer E. A PCR marker linked to a THCA synthase polymorphism is a reliable tool to discriminate potentially THC-rich plants of *Cannabis sativa* L. J Forensic Sci. 2014; 59:919–926. 10.1111/1556-4029.12448 24579739

[16] Tipparat P, Natakankitkul S, Chamnivikaipong P, Chutiwat S. Characteristics of cannabinoids composition of Cannabis plants grown in Northern Thailand and its forensic application. Forensic Sci Int. 2012; 215: 164–170. 10.1016/j.forsciint.2011.05.006 21636228

[17] Cabezudo B, Recio M, Sanchez-Laulhe JM, Del Mar Trigo M, Toro FJ, Polvorinos F. Atmospheric transportation of marijuana pollen from North Africa to the southwest of Europe. Atmos Environ. 1997; 31: 3323–3328.

[18] Coyle HM, Palmbach T, Juliano N, Ladd C, Lee HC. An overview of DNA methods for the identification and individualization of marijuana. Croat Med J. 2003; 44: 315–321. 12808725

[19] de Meijer EPM, Bagatta M, Carboni A, Crucitti P, Moliterni VMC, Ranalli P, et al The inheritance of chemical phenotype in *Cannabis sativa* L. Genetics 2003; 163:335–346. 12586720 10.1093/genetics/163.1.335 PMC1462421

[20] Weiblen GD, Wenger JP, Craft KJ, ElSohly MA, Mehmedic Z, Treiber EL, et al Gene duplication and divergence affecting drug content in *Cannabis sativa*. New Phytol. 2015; 208: 1241–1250. 10.1111/nph.13562 26189495

[21] McKernan KJ, Helbert Y, Tadigotla V, McLaughlin S, Spangler J, Zhang L, et al. Single molecule sequencing of THCA synthase reveals copy number variation in modern drug-type Cannabis sativa L. BioRxiv 2015

[22] van Bakel H, Stout J, Cote A, Tallon C, Sharpe A, Hughes T, et al The draft genome and transcriptome of *Cannabis sativa*. Genome Biol. 2011; 12: R102 10.1186/gb-2011-12-10-r102 22014239 PMC3359589

[23] Sawler J, Stout JM, Gardner KM, Hudson D, Vidmar J, Butler L, et al The genetic structure of marijuana and hemp. 2015; PLoS ONE 10: e0133292 10.1371/journal.pone.0133292 26308334 PMC4550350

[24] Alghanim HJ, Almirall JR. Development of microsatellite markers in *Cannabis sativa* for DNA typing and genetic relatedness analyses. Anal. Bioanal. Chem. 2003; 376: 1225–1233. 10.1007/s00216-003-1984-0 12811461

[25] Gilmore S, Peakall R, Robertson J. Short tandem repeat (STR) DNA markers are hypervariable and informative in *Cannabis sativa*: implications for forensic investigations. Forensic Sci Int. 2003; 131: 65–74. 12505473 10.1016/s0379-0738(02)00397-3

[26] Hsieh HM, Hou RJ, Tasi LC, Wei CS, Liy SW, Huang LH, et al A highly polymorphic STR locus in *Cannabis sativa*. Forensic Sci Int. 2003; 131: 53–58. 12505471 10.1016/s0379-0738(02)00395-x

[27] Howard C, Gilmore S, Robertson J, Peakall R. Developmental validation of a *Cannabis sativa* STR multiplex system for forensic analysis. J Forensic Sci. 2008; 53: 1061–1067. 10.1111/j.1556-4029.2008.00792.x 18624889

[28] Howard C, Gilmore S, Roberston J, Peakall R. A *Cannabis sativa* STR genotype database for Australian seizures: Forensic applications and limitations. J Forensic Sci. 2009; 54: 556–563. 10.1111/j.1556-4029.2009.01014.x 19302382

[29] Köhnemann S, Nedele J, Schwotzer D, Morzfeld J, Pfeiffer H. The validation of a 15 STR multiplex PCR for *Cannabis* species. Int J Legal Med. 2012; 126: 601–606. 10.1007/s00414-012-0706-6 22573357

[30] Gao C, Xin P, Cheng C, Tang Q, Chen P, Wang C, et al Diversity analysis in *Cannabis sativa* based on large-scale development of expressed sequence tag-derived simple sequence repeat markers. PLoS ONE. 2012; 9: e110638. 10.1371/journal.pone.0110638 PMC4203809 25329551

[31] Chandra S, Lata H, Techen N, Mehmadic Z, Khan IA, ElSolhly MA. Analysis of genetic diversity using SSR markers and Cannabinoid contents in different varieties of *Cannabis sativa* L. Planta Med. 2011; 77-P_5.

[32] Valverde L, Lischka C, Scheiper S, Nedele J, Challis R, de Pancorbo MM, et al Characterization of 15 STR cannabis loci: nomenclature proposal and SNPSTR haplotypes. Forensic Sci Int Genet. 2014a; 9: 61–65. 24528581 10.1016/j.fsigen.2013.11.001

[33] Valverde L, Lischka C, Erlemann S, de Meijer E, de Pancorbo MM, Pfeiffer H, et al Nomenclature proposal and SNPSTR haplotypes for 7 new *Cannabis sativa* L. STR loci. Forensic Sci Int Genet. 2014b; 13: 185–186. 25173491 10.1016/j.fsigen.2014.08.002

[34] Presinszka M, Stiasna K, Vyhnanek T, Trojan V, Mrkvicova E, Hrivna L, et al. Analysis of microsatellite markers in hemp (Cannabis sativa L.). MendelNet Conference 2015; 434–438.

[35] Houston R, Birck M, Hughes-Stamm S, Gangitano D. Evaluation of a 13-loci STR multiplex system for Cannabis sativa genetic identification. Int J Legal Med. 2016; 130: 635–647. 10.1007/s00414-015-1296-x 26661945

[36] Piluzza G, Delogu G, Cabras A, Marceddu S, Bullitta S. Differentiation between fiber and drug types of hemp (*Cannabis sativa* L.) from a collection of wild and domesticated accessions. Genet Resour Crop Evol. 2013; 60: 2331–2342.

[37] Jombart T. *adegenet*: a R package for the multivariate analysis of genetic markers. Bioinformatics. 2008; 24: 1403–1405. 10.1093/bioinformatics/btn129 18397895

[38] Pritchard JK, Stephens M, Donnelly P. Inference of population structure using multilocus genotype data. Genetics. 2000; 155: 945–959. 10835412 10.1093/genetics/155.2.945 PMC1461096

[39] Evanno G, Regnaut S, Goudet J. Detecting the number of clusters of individuals using the software STRUCTURE: a simulation study. Mol Ecol. 2005; 14: 2611–2620. 10.1111/j.1365-294X.2005.02553.x 15969739

[40] Earl DA, vonHoldt BM. STRUCTURE HARVESTER: a website and program for visualizing STRUCTURE output and implementing the Evanno method. Conserv Genet Resour. 2012; 4: 359–361.

[41] Jakobsson M, Rosenberg NA. CLUMPP: a cluster matching and permutation program dealing with label switching and multimodality in analysis of population structure. Bioinformatics. 2007; 23: 1801–1806. 10.1093/bioinformatics/btm233 17485429

[42] Rosenberg NA. distruct: a program for the graphical display of population structure. Mol Ecol Notes. 2004; 4: 137–138.

[43] Goudet J. FSTAT (Version 1.2): a computer program to calculate F-statistics. J Hered. 1995; 86:485–486.

[44] Peakall R, Smouse PE. GenAlEx 6.5: genetic analysis in Excel. Population genetic software for teaching and research-an update. Bioinformatics. 2012; 28: 2537–2539. 10.1093/bioinformatics/bts460 22820204 PMC3463245

[45] Piry S, Alapetite A, Cornuet JM, Paetkau D, Baudouin L, Estoup A. GeneClass2: A software for genetic assignment and first-generation migrant detection. J Hered. 2004; 95: 536–539. 10.1093/jhered/esh074 15475402

[46] Rannala B, Mountain JL. Detecting immigration by using multilocus genotypes. Proc Natl Acad Sci USA. 1997; 94: 9197–9201. 9256459 10.1073/pnas.94.17.9197 PMC23111

[47] Paetkau D, Slade R, Burden M, Estoup A. Genetic assignment methods for the direct, real-time estimation of migration rate: a simulation-based exploration of accuracy and power. Mol Ecol. 2004; 13: 55–65. 14653788 10.1046/j.1365-294x.2004.02008.x

