# Supplementary figures and images for: Broad-Scale Genetic Diversity of Cannabis for Forensic Applications
Source: PLoS One. 2017 Jan 20;12(1):e0170522. doi: 10.1371/journal.pone.0170522 (PMC5249207; doi:10.1371/journal.pone.0170522)

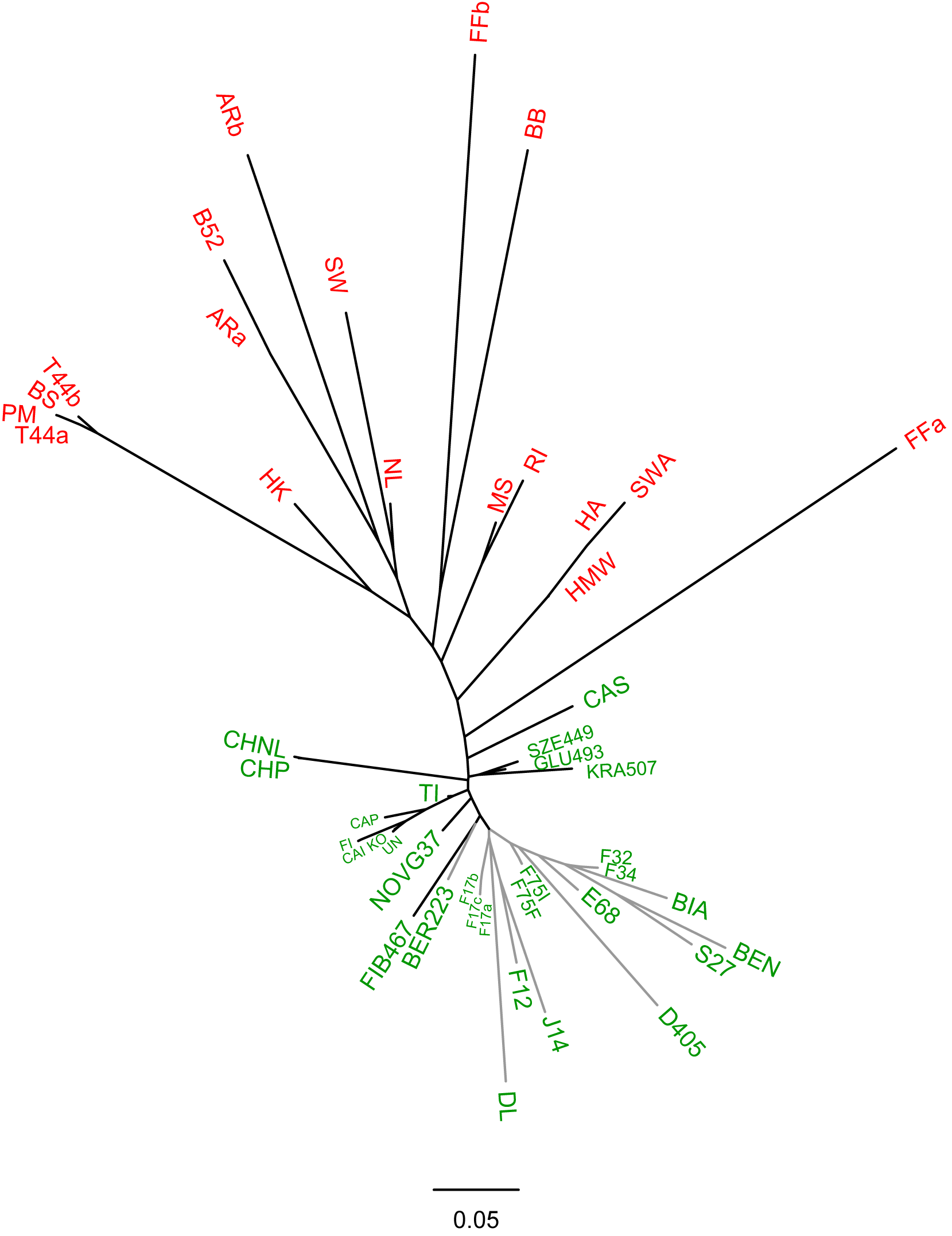

Supplement: S1 Fig — Monoecious hemp are highlighted in grey. (TIF) [file pone.0170522.s001.tif]

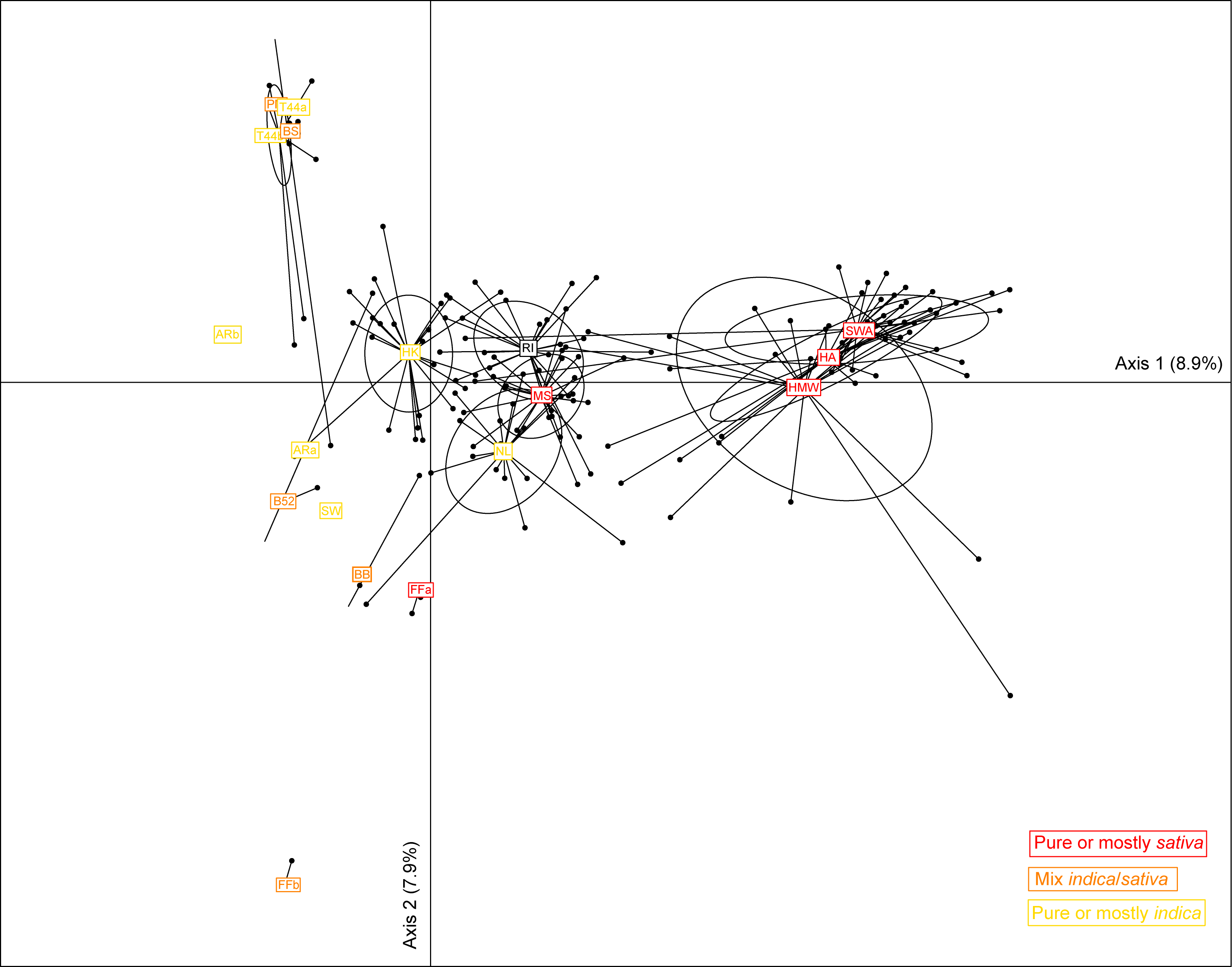

Supplement: S2 Fig — (TIF) [file pone.0170522.s002.tif]
